# Normal appearances and dimensions of the foetal cavum septi pellucidi and vergae on in utero MR imaging

**DOI:** 10.1007/s00234-020-02364-5

**Published:** 2020-01-30

**Authors:** Deborah Jarvis, Paul David Griffiths

**Affiliations:** Academic Unit of Radiology, University of Sheffield, Floor C, Royal Hallamshire Hospital, Glossop Road, Sheffield, S10 2JF UK

**Keywords:** Foetal, Cavum septi pellucidi and vergae, MR imaging

## Abstract

**Purpose:**

The aim of this study is to provide normative data about the appearances and dimensions of the cavum septi pellucidi and vergae (CSPV) on in utero MR (iuMR) imaging in second and third trimester foetuses.

**Methods:**

Two hundred normal foetuses (from a low-risk pregnancy, with normal ante-natal USS findings and no intracranial abnormality of iuMR) had iuMR imaging between 18 and 37 gestational weeks (gw). The anatomical features on those studies were compared with published atlases of post-mortem foetal brains. The length, width and volume of the CSPV were measured in all foetuses.

**Results:**

The anatomy of the CSPV and its relationship with the corpus callosum and the fornices on iuMR imaging was comparable with post-mortem data at all gestational ages studied. The length of the CSPV increased throughout pregnancy, whereas the width and volume of CSPV reached a maximum between 29 and 31 gw and then showed a reduction later in pregnancy.

**Conclusion:**

The iuMR imaging features of the CSPV and its close anatomical relations closely correspond to post-mortem data. The CSPV was patent in all cases but we have shown that closure commences in the midpart of the third trimester and advances in a posterior to anterior direction.

**Electronic supplementary material:**

The online version of this article (10.1007/s00234-020-02364-5) contains supplementary material, which is available to authorized users.

## Introduction

This paper concerns the appearances of the septum pellucidum and septum vergae in foetuses as shown on in utero magnetic resonance (iuMR) imaging. The terminology of these structures has been debated in the literature. The addition of ‘cavum’ because of the cavity that exists between the septa in the foetus gives rise to confusion in parsing the Latin neuter noun [1, 2] so, for simplicity, we refer to ‘cavum septi pellucidi’ and ‘cavum vergae’ as the abbreviation ‘CSPV’ to indicate the paired leaflets of the septa and the enclosed cavities. Where we need to distinguish between the septa and the cavities in foetuses, we will do so in full and we use ‘septum pellucidum’ to indicate the normal combined structure in children/adults.

The septum pellucidum is used as an indicator of normal development of the midline structures of the cerebral hemispheres on post-natal neuroimaging studies [3–6] and visualisation of the CSPV is an essential part of the ante-natal assessment of the foetal brain using either ultrasonography (USS) [7] or iuMR imaging. Its importance is explained by its proximity to, and inter-related development with, functionally important structures such as the corpus callosum, fornices and anterior commissure. Accordingly, the CSPV is an exceptionally important anatomical landmark in foetal neuroimaging, despite the observation that little, or no, clinical function can be attributed to the septum pellucidum after birth.

In this paper, we review the expected anatomy of the CSPV and its adjacent structures from published atlases of post-mortem foetal brains and compare those with observations from 200 iuMR imaging studies of normal second and third trimester foetuses. We will also present data concerning linear and volume measurements of the CSPV in normal foetuses using 3D volume acquisitions from iuMR.

## Methods

### Post-mortem anatomy of the foetal CSPV and related structures

**Fig. 1:**
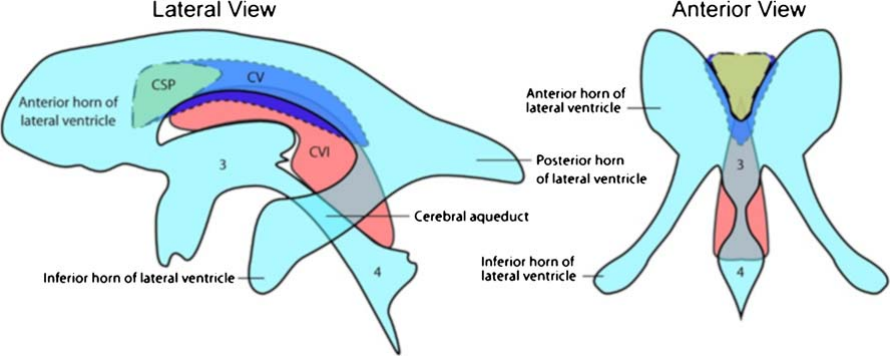
Lateral and frontal line diagrams of the cavum septi pellucidi (green) and cavum vergae (blue) and their anatomical relationship with the ventricular system. The cavum velum interpositum is shown in red (CVI). Taken from: Cavum septum pellucidum. DJ Bell, B Amini et al. Radiopaedia rID: 1066

**Fig. 2:**
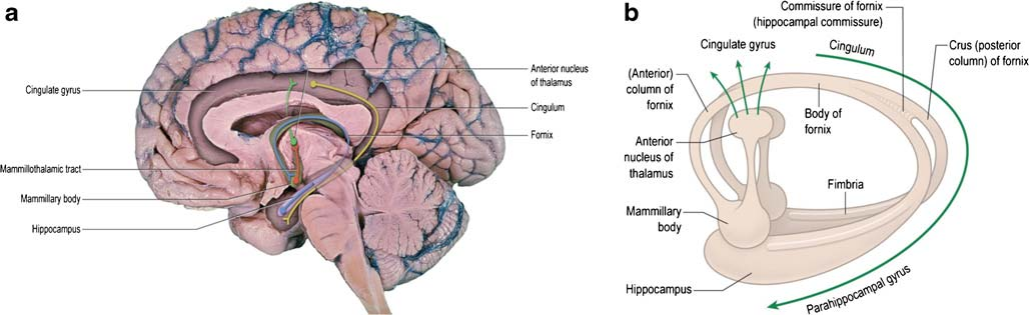
Images to show the anatomy and connectivity of the fornices. **a** An image of the medial part of the right hemisphere overlain with the path of the fibres that constitute the fornices in blue. Those neurons start as the fimbria in the hippocampus and terminate in the mammillary bodies (pre-commissural fibres only) and synapse with neurons passing to the thalamus as the mammillothalamic tract (of Vicq d’Azyr), which is shown in red. This is shown diagrammatically in **b**. **a** shows that the major afferent to the hippocampus, in terms of the fornix, is from the cingulate gyrus and that structure is the ultimate destination of the thalamic neurons controlled by the neurons in the mammillothalamic tract (in green). This constitutes the functionally important circuit of Papez, which is part of the limbic system. From Neuroanatomy: An Illustrated Colour Text; 6e (AR Crossman and D Neary), figures by Ben Crossman, courtesy of Elsevier

The normal anatomy of the foetal CSPV and its close neighbours (corpus callosum and fornices) was reviewed from four atlases of post-mortem sections during the second and third trimesters [8–11]. The close relationship between the CSPV and the lateral and third ventricles and its relationship with the fornix are shown diagrammatically in Figs. 1 and 2.

### Ethical approval and recruitment for the iuMR imaging studies

iuMR imaging studies of the brain were performed on 200 normal foetuses at our institution between 2014 and 2017 as part of a wider study of the use of iuMR for showing foetal neuropathology [12, 13]. Normality was defined as a foetus from a low-risk pregnancy, with normal ante-natal USS findings (whole body) and no intracranial abnormality of iuMR. Post-natal neurodevelopmental assessments were not undertaken. All of the pregnant women provided written, informed consent under the guidance and with the approval of a national Ethics Committee.

### In utero MR imaging technique and analysis

All of the iuMR studies were performed on the same whole body 1.5 T scanner (HDx - General Electric Healthcare, Waukesha, WI) using flexible phased-array body coils. Our standard imaging protocol consisted of single-shot fast spin echo (ssFSE) T2-weighted sequences in the three orthogonal planes, axial ultrafast T1-weighted imaging and diffusion-weighted imaging in the axial planes. All of the foetuses also received 3D volume imaging using steady-state methods as described in detail elsewhere [14]. The iuMR studies were reported at the time the examinations were performed by a paediatric neuroradiologist with > 18 years’ experience of foetal neuroimaging (PDG) and all were confirmed as structurally normal. For the purposes of this study, all of the examinations were reviewed by the authors in order to look for structural variations of the CSPV, corpus callosum and fornices.

**Fig. 3:**
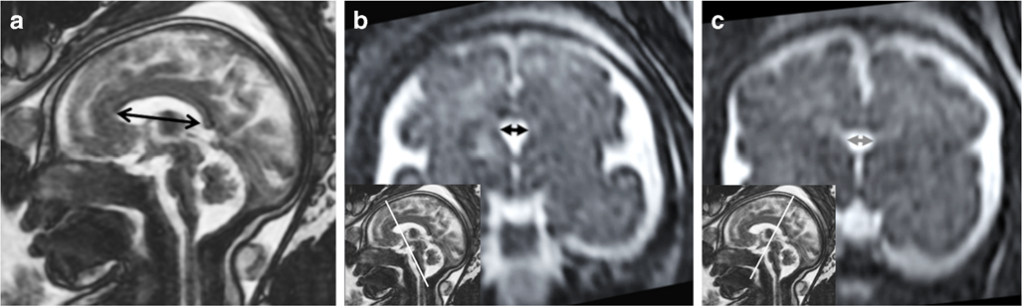
Reconstructed images from T2-weighted volume imaging of a foetal brain at 31 gw. **a** The midline sagittal image showing how the cranio-caudal length of the CSPV was measured (double-headed arrow). **b** A coronal image showing how the coronal width of the cavum septi pellucidi was measured (double-headed arrow on **b**) with an inset to show the coronal plane used. **c** The equivalent images for measuring the width of the cavum vergae

The length and width of the foetal CSPV were measured on coronal and sagittal reformatted images produced from the T2-weighted 3D volume acquisitions. Proprietary software (Volume Viewer, General Electric Healthcare, Waukesha, WI) was used to create a midline sagittal image with a slice partition identical to the base data (1.0–1.3 mm). The cranio-caudal dimension of the CSPV was measured from the inner surface of the genu to the inner surface of the splenium of the corpus callosum. The maximum widths of the cavum septum pellucidum and cavum vergae were measured separately on coronal reformations from the T2-weighted 3D volume acquisitions by measuring the distance between the two inner borders of the septa of the CSPV (Fig. 3). We used an arbitrary delineation of the boundary between the cavum septi pellucidi and the cavum vergae as the anterior margin of the thalamus. We accept that the usual distinction is made by the relationship of the cavities to the division of the body of the fornix into the anterior columns but this feature is not seen reliably on iuMR studies because of limitations in anatomical and contrast resolution.

Measurements of the widths of the cavum septi pellucidi and cavum vergae and the length and volume of the CSPV are presented by the week of gestational age in terms of mean, standard deviation and full range. The whole datasets for each parameter were plotted against gestational age and a regression analysis was performed using Microsoft Excel (2016) in order to produce a trend line and its regression equation. The best fit for the trend line was chosen based on the highest *R*^2^ value, determined by successive analysis of polynomial fits (linear, quadratic and cubic).

## Results

### Anatomy of the foetal CSPV and related structures

**Fig. 4:**
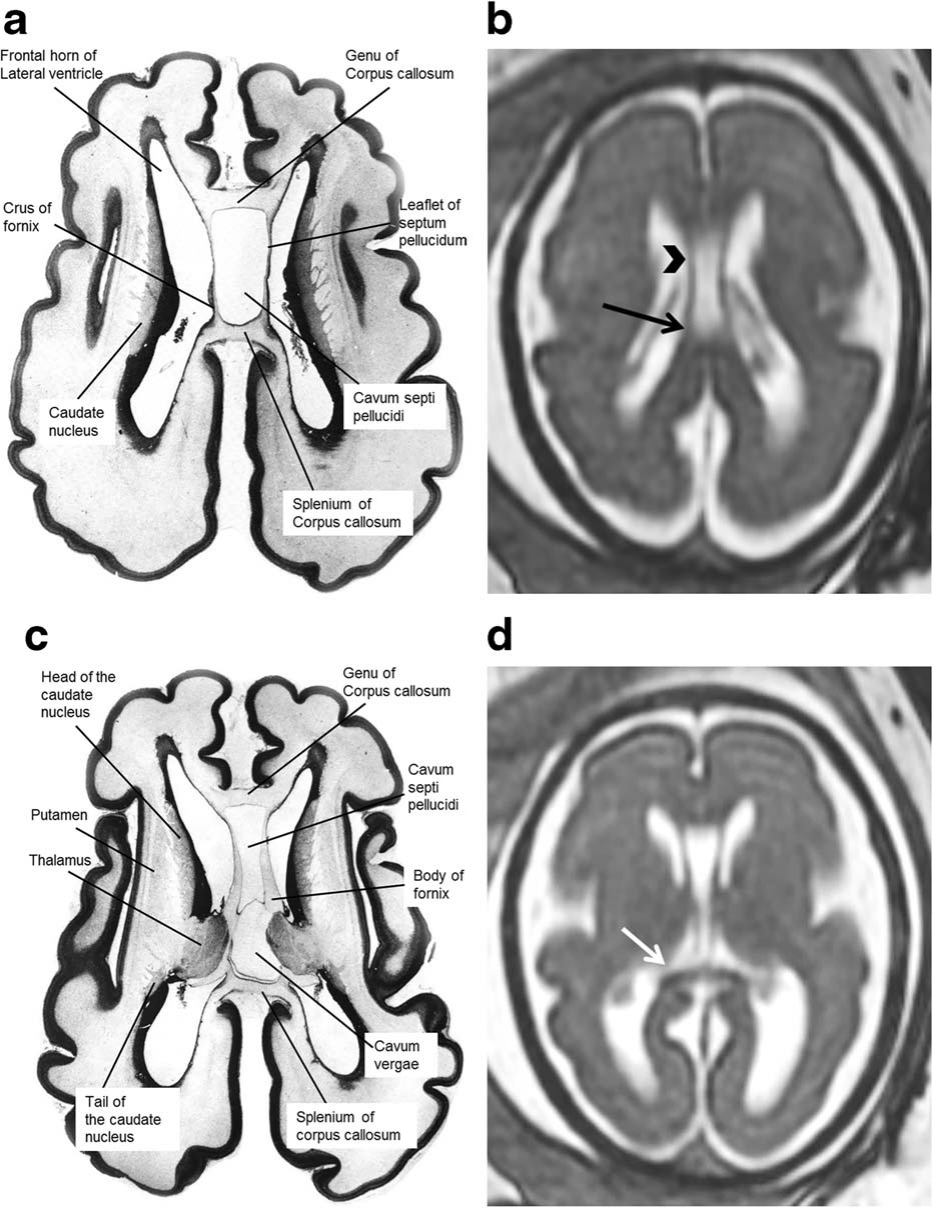

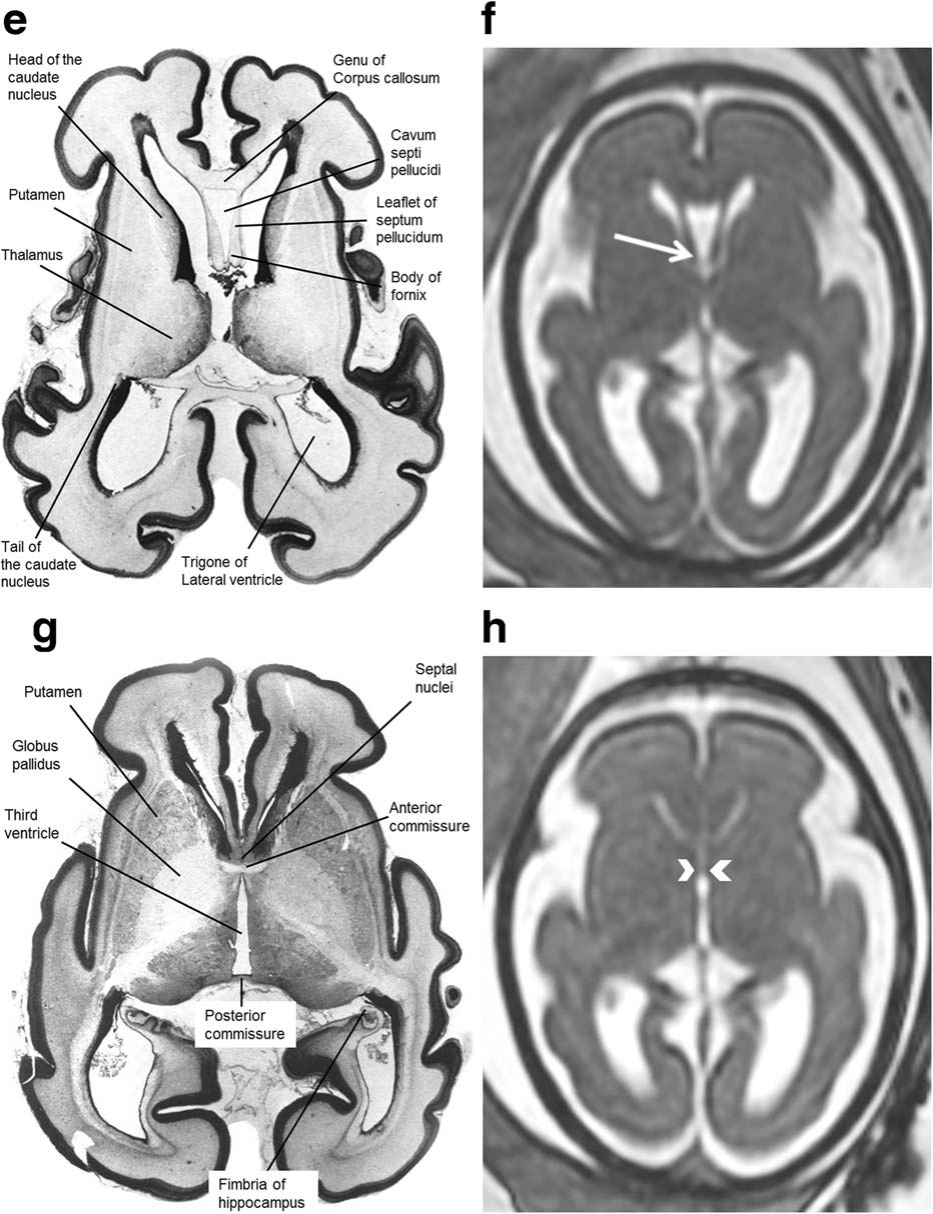
Axial post-mortem specimens (**a**, **c**, **e**, **g**—with anatomical annotation modified from reference [9]) with equivalent axial reconstructions from T2W volume acquisitions (**b**, **d**, **f**, **g**) from 29 gw foetuses running from superior to inferior. Images in this plane show the crura of the fornices originating from the hippocampus and heading towards the splenium of the corpus callosum (arrowed on **b** and **d**) with the leaflets of the CSPV anteriorly (arrowheads on **b**). The fornices course anteriorly and are in close proximity (arrowed on **f**) before dividing around the anterior commissure (arrowheads on **h**)

**Fig. 5:**
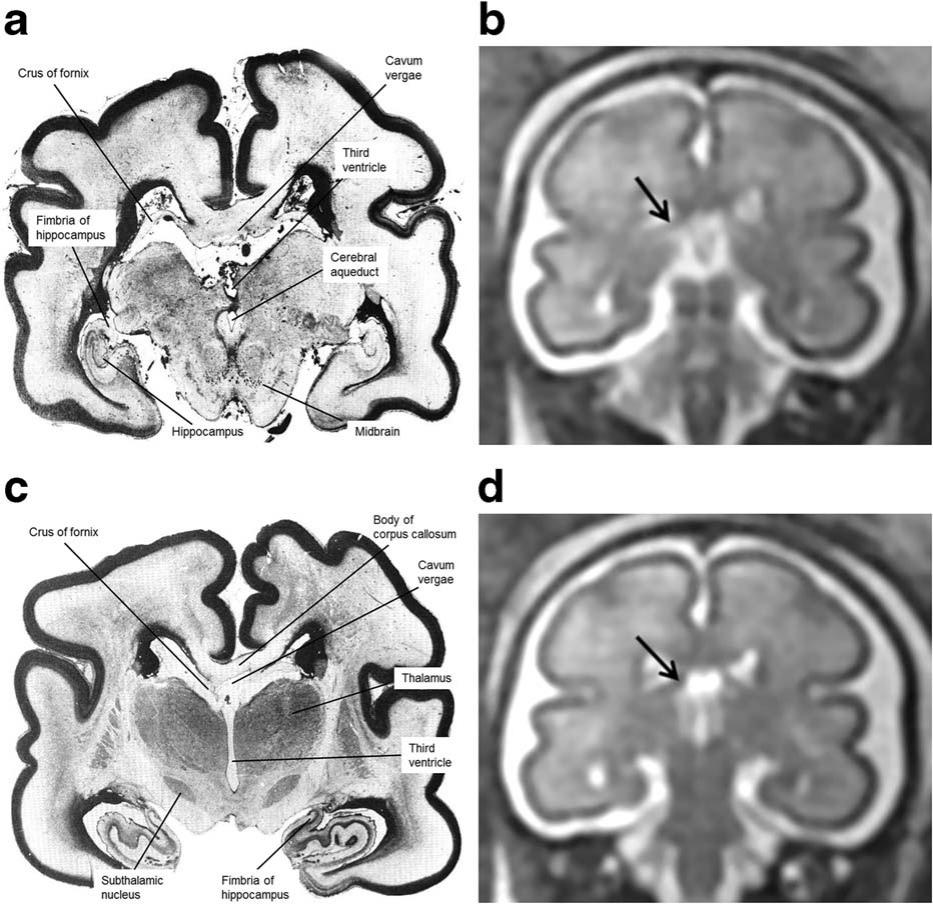

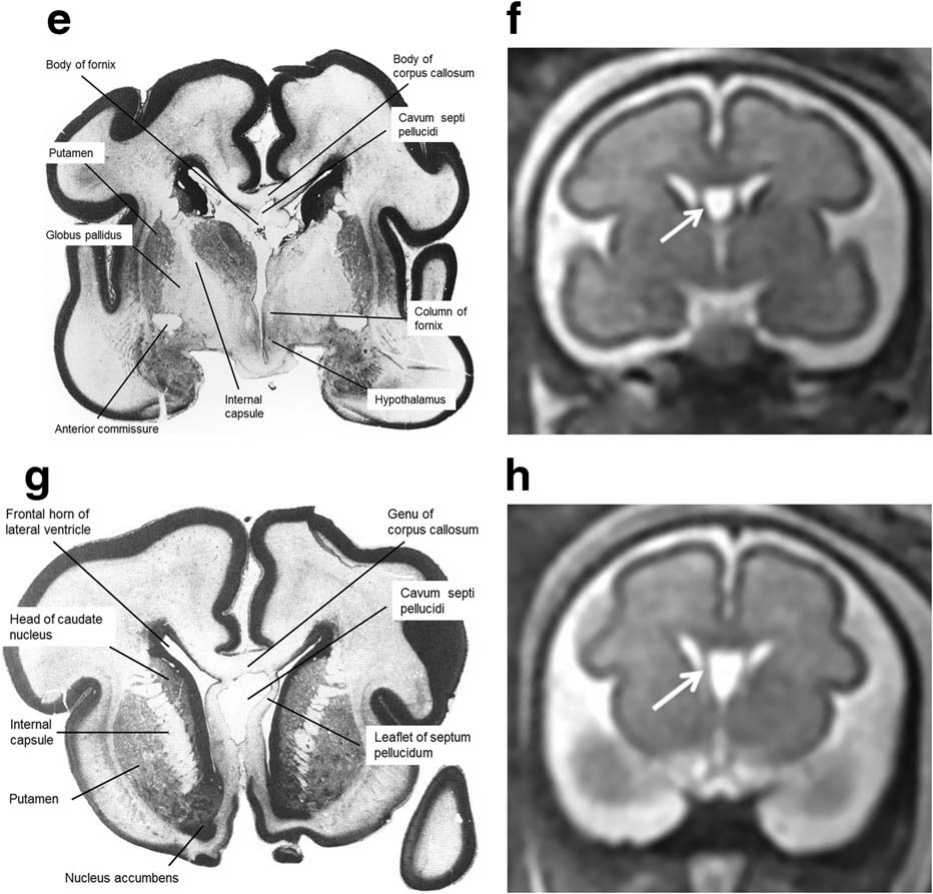
Coronal post-mortem specimens (**a**, **c**, **e**, **g**—with anatomical annotation modified from reference [9]) and equivalent coronal T2W single-shot fast spin echo images (**b**, **d**, **e**, **f**) from 29 gw foetuses running from caudal to cranial. Images in this plane show the crura of the fornices originating from the fimbria of the hippocampi (arrowed on **b**) and heading towards the splenium of the corpus callosum (arrowed on **d**) and the relationship between the leaflets of the CSPV and body of the fornices inferiorly (arrowed on **f**). The fornix enters the brain parenchyma in the vicinity of the anterior commissure so the cranial images only show the leaflets of the CSPV (arrowed on **h**)

**Fig. 6:**
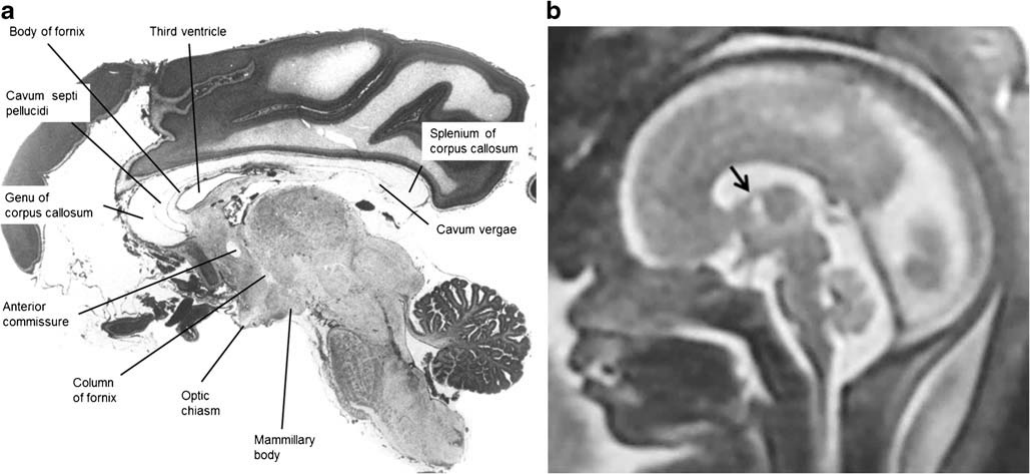
Midline sagittal post-mortem specimen (**a**—with anatomical annotation modified from reference [9]) and midline sagittal T2W single-shot fast spin echo image (**b**) from 29 gw foetuses. Images in this plane show the relationship between the corpus callosum and fornices (body of the fornices arrowed on **b**) therefore defining the fluid-filled central portion of the CSPV. Note the CSPV appears larger on the iuMR imaging, presumably because of loss of fluid pressure post-mortem

The anatomy of the CSPV is understood by appreciating its relationship with the corpus callosum and fornices and there was remarkable consistency between the anatomy of those structures shown on atlases of post-mortem sections and on the iuMR imaging from normal foetuses reported here (Figs. 4, 5 and 6). As expected, the corpus callosum appeared fully formed on iuMR imaging in all 200 cases between 18 and 37 gestational weeks (gw), inasmuch as its full cranio-caudal extent had been achieved and its three major anatomical subdivisions (genu, body and splenium) could all be recognised routinely. The inferior border of the corpus callosum indicates the superior extent of the CSPV. Inferiorly, the origin of the fornices from the fimbriae (the major efferent pathway of the hippocampus) can be seen, although there is insufficient spatial and contrast resolution to separate the fimbria from the rest of the mesial temporal lobe on iuMR imaging. As such, the posterior part of the fornices (crura) can be seen exiting from the posterior part of the mesial temporal lobe and pass towards the splenium of the corpus callosum. This feature can be demonstrated on iuMR imaging in the majority of foetuses studied (Figs. 4 and 5), particularly on the thinner partitions available from volume data sets reconstructed in the coronal plane. The crura of the fornices come into close proximity with its contra-lateral partner in the vicinity of the splenium of the corpus callosum and it is not possible to differentiate the fornices from the splenium of the corpus callosum in this location. The fornices then arc forwards, away from the splenium, and diverge from the body of the corpus callosum but the septa of the CSPV extend between the corpus callosum and fornices. This is best appreciated in the coronal plane (Fig. 5) although the more cranial regions are well seen on sagittal images (Fig. 6). The septa of the CSPV are generally widely separated throughout but inferiorly they come into close proximity where they contact fused bodies of the fornices (Fig. 5).

It is difficult to delineate where the anterior portion of the body of the fornices divide into the columns of the fornices on iuMR imaging, although they can often be seen just superior and posterior to the anterior commissure. The columns of the fornices then separate from the leaflets of the CSPV as they enter brain parenchyma immediately behind the anterior commissure. The passage of the columns of the fornices to the mammillary bodies can be seen on stained histological sections; this detail is not visible on iuMR imaging (Figs. 4 and 5). The anterior parts of the CSPV terminate in contact with the genu of the corpus callosum.

### Dimensions of the CSPV measured on iuMR imaging

**Fig. 7:**
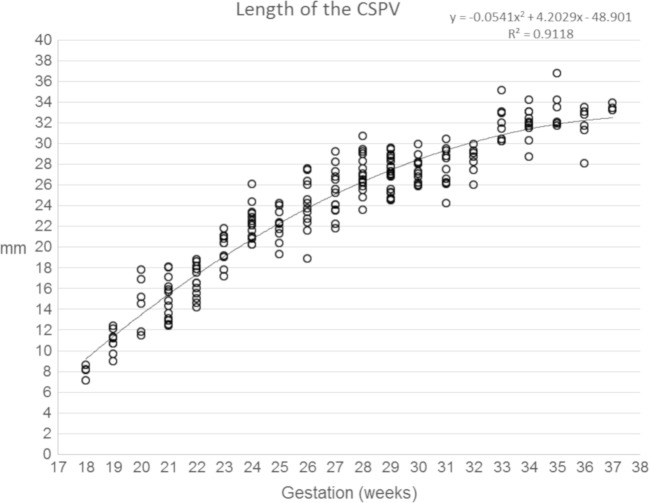
A graph showing the length of the CSPV in 200 normal foetuses in relation to their gestational age. The results are also presented in a tabular form in supplementary material 1. See text for details

**Fig. 8:**
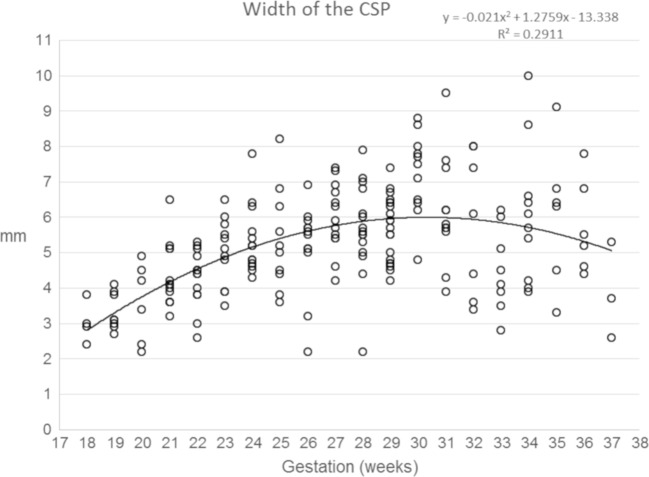
A graph showing the width of the cavum septi pellucidi in 200 normal foetuses in relation to their gestational age. The results are also presented in a tabular form in supplementary material 2. See text for details

**Fig. 9:**
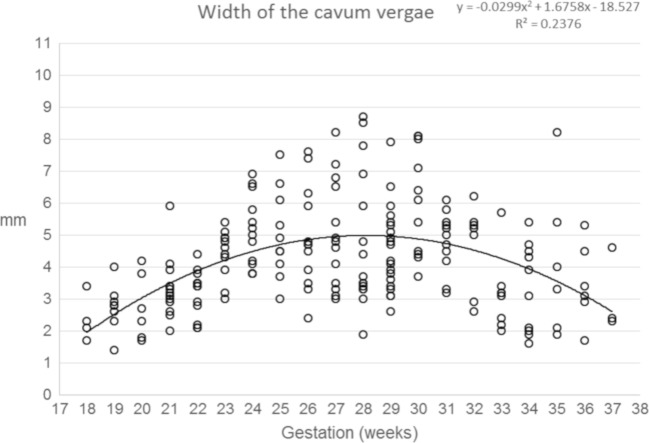
A graph showing the width of the cavum vergae in 200 normal foetuses in relation to their gestational age. The results are also presented in a tabular form in supplementary material 3. See text for details

**Fig. 10:**
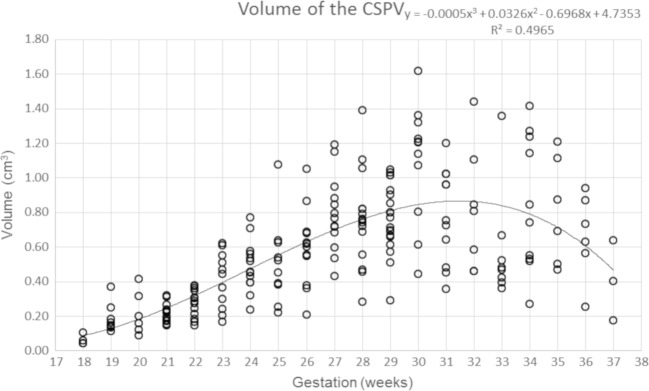
A graph showing the volume of the CSPV in 200 normal foetuses in relation to their gestational age. The results are also presented in a tabular form in supplementary material 4. See text for details

Data concerning the length of the CSPV, the widths of the cavum septi pellucidi and cavum vergae and the volume of fluid within the CSPV are shown in Figs. 7, 8, 9 and 10 and supplementary materials 1–4. There was a strong, positive correlation between the length of the CSPV and gestational age, which was best described by a quadratic function as shown in Fig. 7. The increasing length of the CSPV throughout pregnancy is likely to reflect the rapid expansion of the cerebral hemispheres in the second and third trimesters. The width of the cavum septi pellucidi was greater than the width of the cavum vergae at every gestational stage with both structures attaining their maximum width around 29–31 gw (Figs. 8 and 9). After that time, there was a reduction in width, most prominently in the cavum vergae, although a cavity was present in all foetuses in the study. Those features are likely to represent commencement of the closure of the CSPV, which is usually complete after birth. The fluid in the cavity of the CSPV showed a similar reduction in volume after 31 gw (Fig. 10).

The cavum velum interpositum was not a subject for our study but we noted that it was visualised in several foetuses but it was not possible to make robust measurements because of its small size. No further comment is made about that structure in this paper.

## Discussion

We have reviewed the anatomy of the normal septum pellucidum on MR imaging in children in an earlier publication [6] and post-natally, the normal septum pellucidum has a highly consistent appearance as a thin, vertically orientated structure that forms the medial border of the frontal horns and bodies of the lateral ventricles. It extends between the corpus callosum superiorly and the fornices inferiorly. Its cranial extent is the rostrum and genu of the corpus callosum and caudal extension, the septum vergae, extends posteriorly to the splenium of the corpus callosum. Histologically, the normal septum pellucidum in children and adults consists of two fused leaves (1–3 mm thick) with a thin, fluid-containing space that is extra-pial and as a result it does not communicate with the ventricular system or extra-axial CSF spaces.

There are no known long-term, consistent clinical sequelae related to disruption of a previously normally formed septum pellucidum. This is in spite of the close proximity to important relationship to structures involved in the Papez circuit of the limbic system (Fig. 2) and the recent description of small numbers of neurons in the leaflets of the mature septum pellucidum [15]. In contrast, non-formation of the septum pellucidum is associated with a relatively high risk of poor neuro-developmental outcome, even if no other developmental brain abnormalities are present. So, it is the detection of a normal CSPV that makes it a highly important structure to define on ante-natal imaging and it is non-visualisation of the CSPV that causes diagnostic concern. A recent publication, however, has raised concern about ‘prominent’ (i.e. persistent and wide) septum pellucidum in young adults [16] and in foetuses [17].

Most published reports about the normal foetal CSPV to date are based on ultrasound and it is reported that the normal CSPV is robustly demonstrated on that modality. Ho et al. [17] reviewed the recent literature and quote three papers [18–20] that report visualisation of CSPV in close to 100% of cases although the inability to visualise the associated anatomical structures (fornices and corpus callosum) consistently is well recognised. It is likely, on theoretical grounds, that iuMR imaging will improve definition of the CSPV and extend visualisation to include robust recognition of the fornices and corpus callosum. This is on the basis of both the ability to image in the three natural orthogonal planes directly with iuMR and the advantage of improved contrast resolution between those brain structures and the adjacent CSF. In our current paper, we have reviewed the anatomy of the CSPV and its neighbouring structures as published in specialist atlases of post-mortem tissue from normal foetuses [8–11] and compared that information with their appearances iuMR imaging of 200 normal foetuses with ages ranging between 18 and 37 gw. The theoretical supposition of improved visualisation on iuMR is supported by our findings in normal foetuses, which shows that the septa and cavity of the CSPV, the fornices and the corpus callosum can be resolved routinely and clearly from the surrounding CSF.

We have described the complicated anatomical relationship between the CSPV, corpus callosum and the fornices in the “Results” section and have concluded that the appearances of the gross anatomy those structures on iuMR imaging were coherent with the specimens in post-mortem atlases over the gestational age range studied. Both the anterior (septum pellucidum) and the posterior (vergae) portions of the CSPV had a fluid-containing cavity in all 200 foetuses, i.e. there was no evidence of fusion of the leaflets in utero earlier than 38 gw. We did show, however, that the dimensions of the CSPV changed with maturation of the foetuses, for example there was a strong positive correlation between the cranio-caudal length of the CSPV and gestational age. This is likely to reflect the rapid growth of the cerebral hemispheres, including the corpus callosum, during the late second and third trimesters.

Alterations in the transverse width of the CSPV with gestational age were more complicated. We measured the widths of the cavum septum pellucidum and the cavum vergae separately and found that they both attained their maximum width around 29–31 gw. This was followed by a reduction in width, which was more pronounced in the cavum vergae, and we interpret this finding as an indication that the closure of the CSPV commences in the mid-third trimester and advances in a posterior to anterior direction. The volume of the CSPV closely followed the pattern of change in width of the CSPV with gestation, inasmuch it reached a maximum between 31 and 32 gw and then reduced.

The normative linear dimensions of width of the CSPV from our study correlate reasonably well with the published measurements made on USS but volumes of the CSPV on USS have not been. The upper limits of the width measurements made on iuMR imaging matched the USS data closely but iuMR generally having smaller lower limits of CSPV width when compared with USS. Our data suggests an upper limit of width of the CSPV at 8–9 mm at any stage of pregnancy, which is in agreement with an older publication that describes the CSPV as a ‘cyst’ if it measures 10 mm or greater. Only 1/200 of the normal foetuses (at 34 gw) in our study had a CSPV width of 10 mm. This is important because of some reports of some adverse clinical significance arising from ‘cystic’ or ‘prominent’, i.e. wide, foetal CSPV [21]. This is not widely accepted at present and requires further research. The corpus callosum was complete in all cases, and in the vast majority of foetuses, the imaging quality was sufficiently high to be able to see the fornices from their posterior origins at the fimbria of the hippocampi to their division around the anterior commissure anteriorly.

In conclusion, we present normative data for the CSPV on iuMR imaging in terms of its appearance and dimensions (length, width and volume) between 18 and 37 gw. We have evidence that the closure of the CSPV commences after 29–31 gw and advances from posterior to anterior.

## Electronic supplementary material

ESM 1 (DOCX 12 kb)

ESM 2 (PNG 181 kb)

High Resolution (TIF 27234 kb)

ESM 3 (PNG 145 kb)

High Resolution (TIF 27067 kb)

ESM 4 (PNG 159 kb)

High Resolution (TIF 27215 kb)

ESM 5 (PNG 157 kb)

High Resolution (TIF 25642 kb)
