# Supplementary material for: Normal appearances and dimensions of the foetal cavum septi pellucidi and vergae on in utero MR imaging
Source: Neuroradiology. 2020 Jan 30;62(5):617–27. doi: 10.1007/s00234-020-02364-5 (PMC7186260; doi:10.1007/s00234-020-02364-5)
Supplement: Supplementary file 1 — (DOCX 12 kb) [file 234_2020_2364_MOESM1_ESM.docx]

**Legends for supplementary material**

**Supplementary Material 1:** Tabulated data of the length of the CSPV in 200 normal fetuses in relation to their gestational age presented as minimum, maximum, mean and standard deviation from 18-37gw. See Figure 7.

**Supplementary Material 2:** Tabulated data of the width of the cavum septi pellucidi in 200 normal fetuses in relation to their gestational age presented as minimum, maximum, mean and standard deviation from 18-37gw. See Figure 8.

**Supplementary Material 3:** Tabulated data of the width of the cavum vergae in 200 normal fetuses in relation to their gestational age presented as minimum, maximum, mean and standard deviation from 18-37gw. See Figure 9.

**Supplementary Material 4:** Tabulated data of the volume of the CSPV in 200 normal fetuses in relation to their gestational age presented as minimum, maximum, mean and standard deviation from 18-37gw. See Figure 10.
