# Supplementary figures and images for: Normal appearances and dimensions of the foetal cavum septi pellucidi and vergae on in utero MR imaging
Source: Neuroradiology. 2020 Jan 30;62(5):617–27. doi: 10.1007/s00234-020-02364-5 (PMC7186260; doi:10.1007/s00234-020-02364-5)

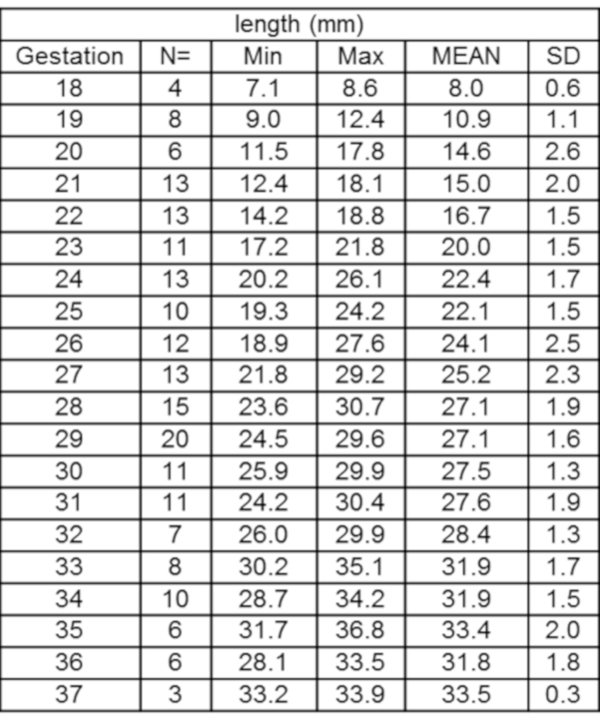

Supplement: Supplementary file 2 — (PNG 181 kb) [file 234_2020_2364_Fig11_ESM.png]

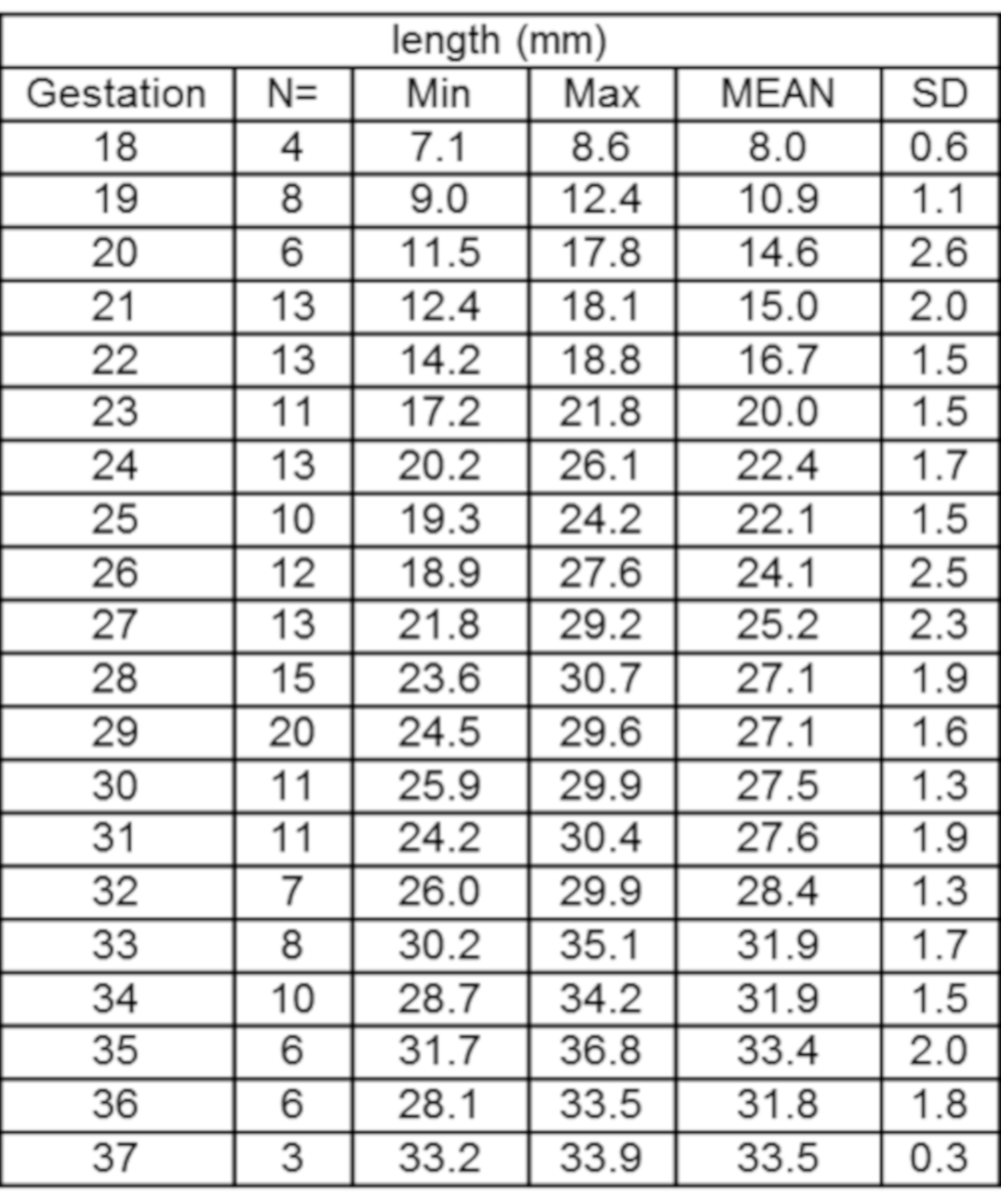

Supplement: Supplementary file 3 — High Resolution (TIF 27234 kb) [file 234_2020_2364_MOESM2_ESM.tif]

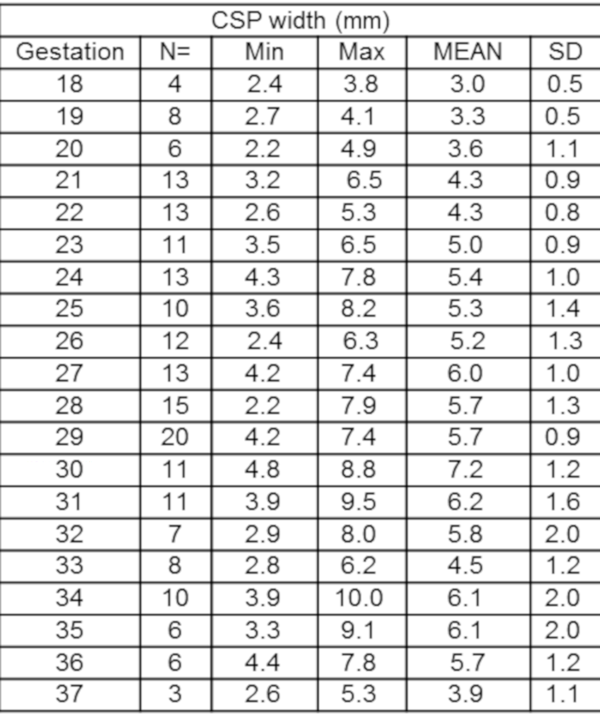

Supplement: Supplementary file 4 — (PNG 145 kb) [file 234_2020_2364_Fig12_ESM.png]

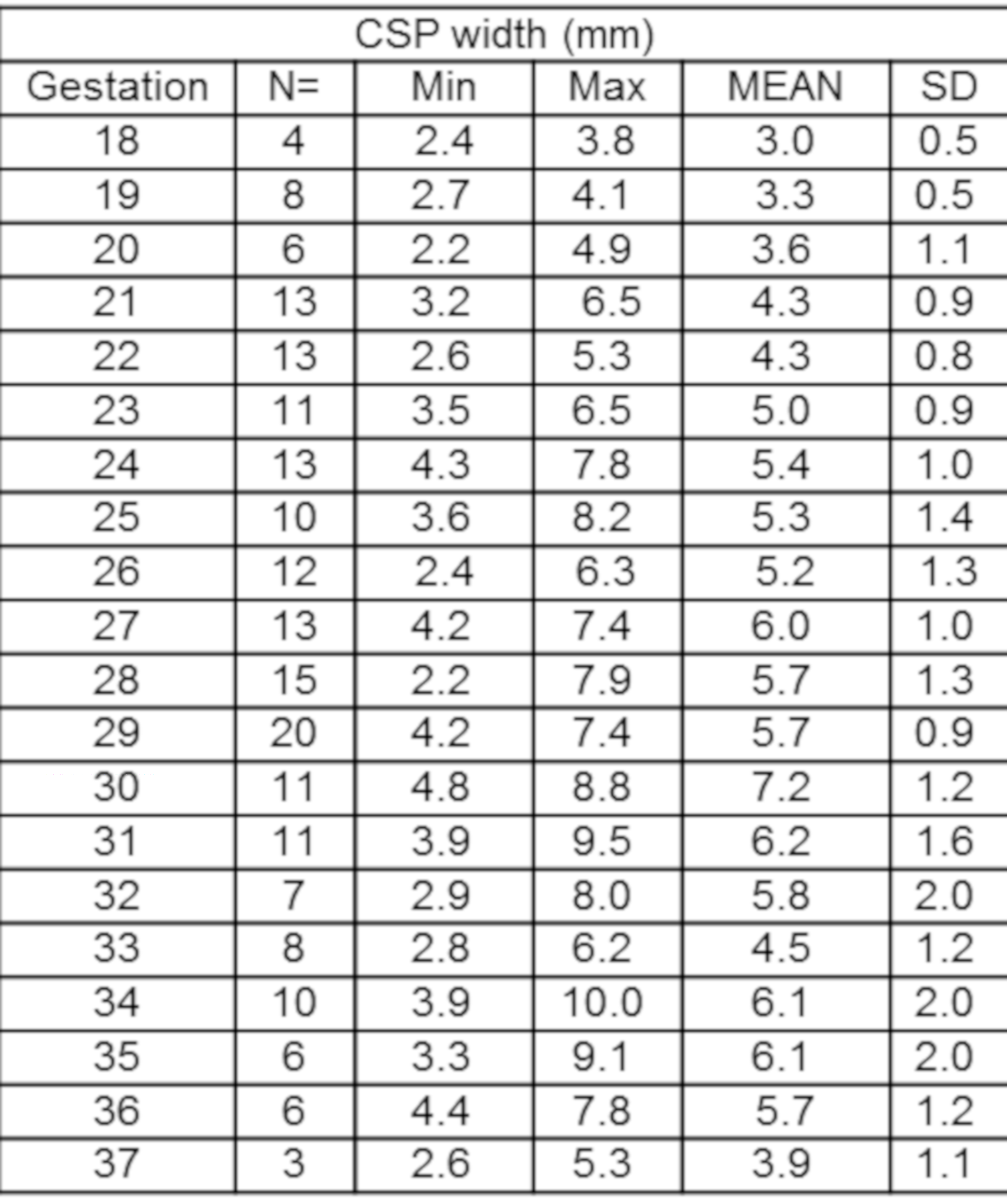

Supplement: Supplementary file 5 — High Resolution (TIF 27067 kb) [file 234_2020_2364_MOESM3_ESM.tif]

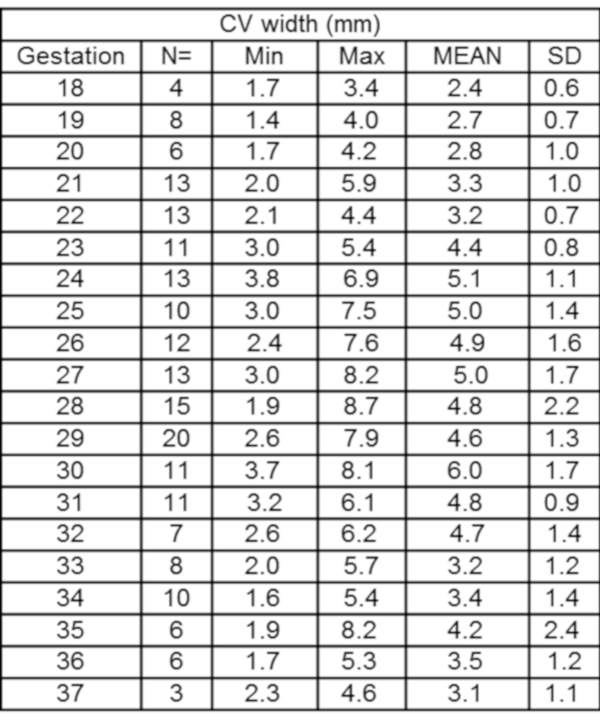

Supplement: Supplementary file 6 — (PNG 159 kb) [file 234_2020_2364_Fig13_ESM.png]

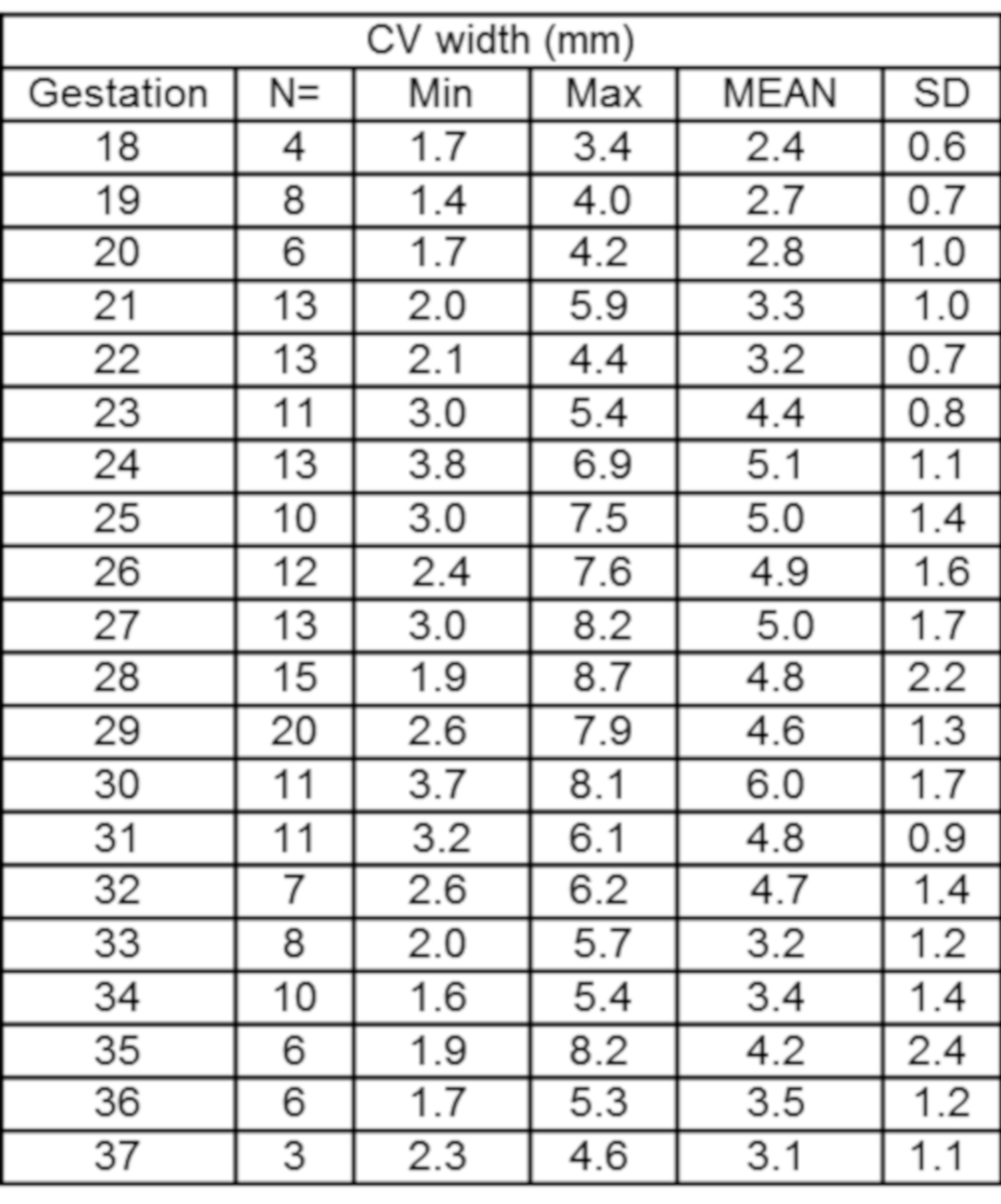

Supplement: Supplementary file 7 — High Resolution (TIF 27215 kb) [file 234_2020_2364_MOESM4_ESM.tif]

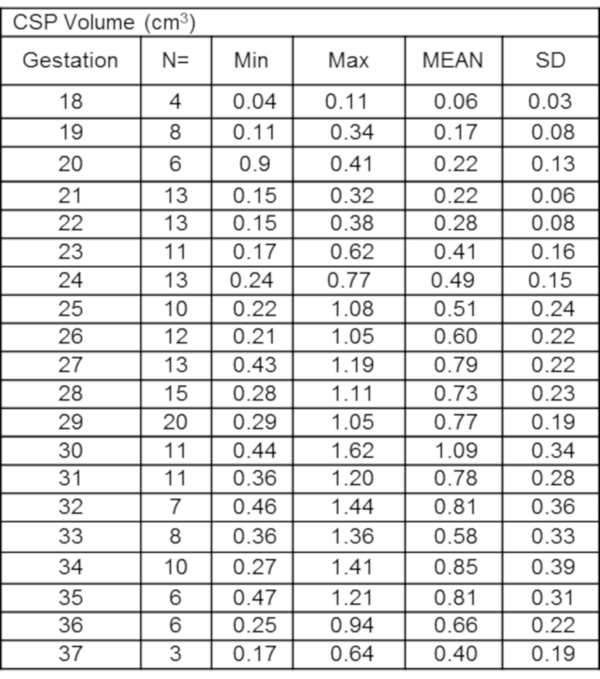

Supplement: Supplementary file 8 — (PNG 157 kb) [file 234_2020_2364_Fig14_ESM.png]

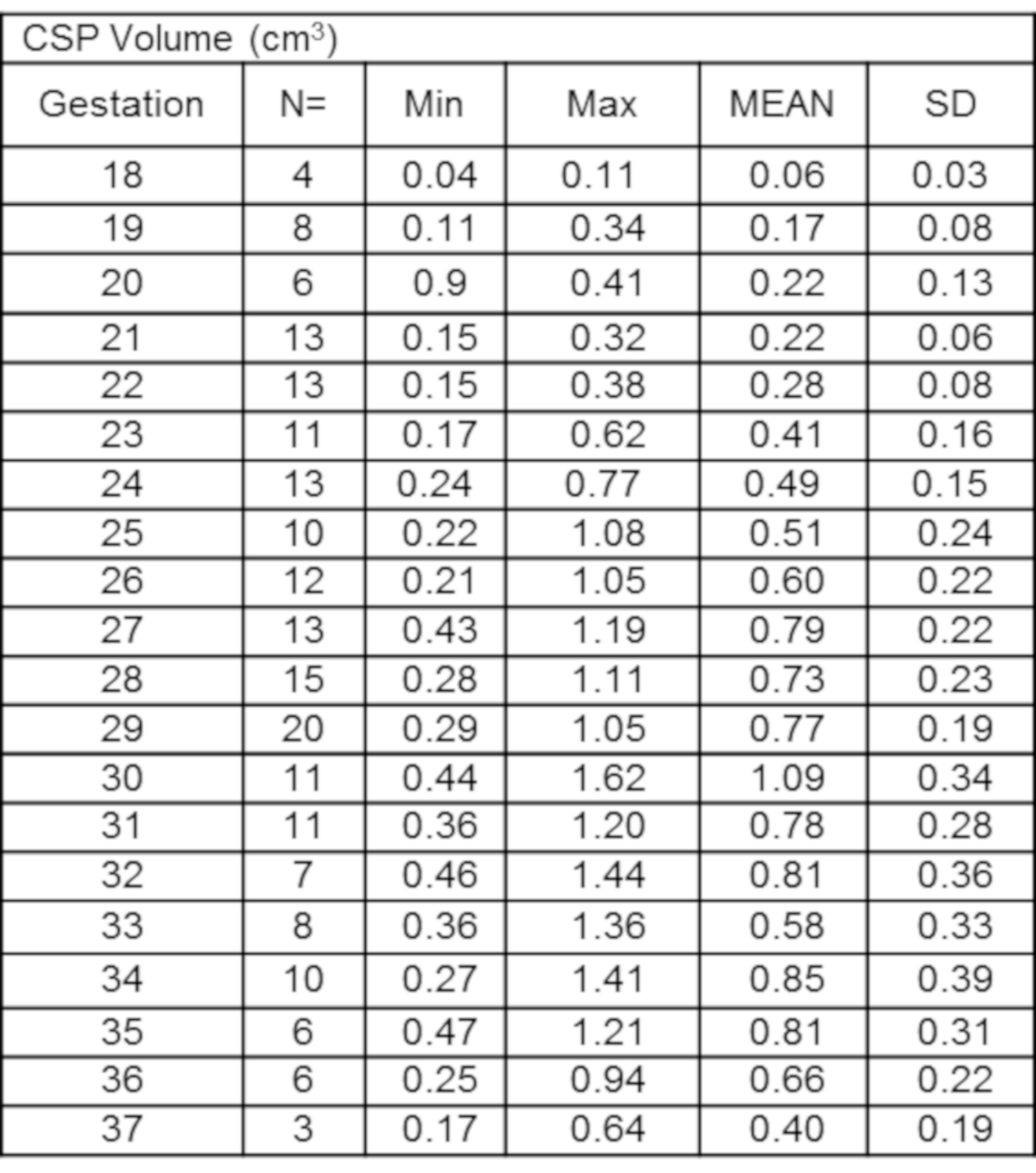

Supplement: Supplementary file 9 — High Resolution (TIF 25642 kb) [file 234_2020_2364_MOESM5_ESM.tif]
